# Rescue of IL-1β-induced reduction of human neurogenesis by omega-3 fatty acids and antidepressants

**DOI:** 10.1016/j.bbi.2017.05.006

**Published:** 2017-10

**Authors:** Alessandra Borsini, Silvia Alboni, Mark A. Horowitz, Luis M. Tojo, Giuseppe Cannazza, Kuan-Pin Su, Carmine M. Pariante, Patricia A. Zunszain

**Affiliations:** a Department of Psychological Medicine, Institute of Psychiatry, Psychology and Neuroscience, King’s College London, UK; b Department of Life Sciences, University of Modena and Reggio Emilia, Modena, Italy; c Department of Psychiatry & Mind-Body Interface Laboratory (MBI-Lab), China Medical University Hospital, College of Medicine, China Medical University, Taichung, Taiwan; d South London and Maudsley NHS Foundation Trust, Denmark Hill, Camberwell, London, UK

**Keywords:** Fish oil, Cytokines, PUFA, Immune, IL-1 beta, Sertraline, Venlafaxine, Neurogenic, Kynurenine-pathway

## Abstract

Both increased inflammation and reduced neurogenesis have been associated with the pathophysiology of major depression. We have previously described how interleukin-1 (IL-1) β, a pro-inflammatory cytokine increased in depressed patients, decreases neurogenesis in human hippocampal progenitor cells. Here, using the same human *in vitro* model, we show how omega-3 (ω-3) polyunsaturated fatty acids and conventional antidepressants reverse this reduction in neurogenesis, while differentially affecting the kynurenine pathway. We allowed neural cells to proliferate for 3 days and further differentiate for 7 days in the presence of IL-1β (10 ng/ml) and either the selective serotonin reuptake inhibitor sertraline (1 µM), the serotonin and norepinephrine reuptake inhibitor venlafaxine (1 µM), or the ω-3 fatty acids eicosapentaenoic acid (EPA, 10 µM) or docosahexaenoic acid (DHA, 10 µM). Co-incubation with each of these compounds reversed the IL-1β-induced reduction in neurogenesis (DCX- and MAP2-positive neurons), indicative of a protective effect. Moreover, EPA and DHA also reversed the IL-1β-induced increase in kynurenine, as well as mRNA levels of indolamine-2,3-dioxygenase (IDO); while DHA and sertraline reverted the IL-1β-induced increase in quinolinic acid and mRNA levels of kynurenine 3-monooxygenase (KMO). Our results show common effects of monoaminergic antidepressants and ω-3 fatty acids on the reduction of neurogenesis caused by IL-1β, but acting through both common and different kynurenine pathway-related mechanisms. Further characterization of their individual properties will be of benefit towards improving a future personalized medicine approach.

## Highlights

• Inflammation and reduced neurogenesis are associated with the pathophysiology of depression.
• IL-1β decreased neurogenesis in human hippocampal progenitor cells.
• EPA, DHA, sertraline and venlafaxine prevented the IL-1β-induced reduction in neurogenesis.
• EPA and DHA reversed the IL-1β-induced increase in kynurenine levels.
• EPA, DHA, sertraline and venlafaxine decreased the upregulation of IDO and KMO mRNA.

## 1 Introduction

Hippocampal neurogenesis has been widely associated with depression: reduced levels have been reported in depressed patients as well as in animal models of depression (Boldrini et al., 2009, Kempermann et al., 2008), and increasing its levels appears sufficient to reduce depressive-like behaviours (Hill et al., 2015). Several factors can regulate neurogenesis: it can be stimulated by antidepressant drugs (Warner-Schmidt and Duman, 2006, Sahay and Hen, 2007, Malberg and Blendy, 2005) and negatively affected by pro-inflammatory cytokines such as interleukin-1 (IL-1) β (Goshen et al., 2008, Koo and Duman, 2008, Kuzumaki et al.). This cytokine is of importance, as increased levels have been shown in peripheral blood and cerebrospinal fluid of depressed patients (Capuron and Miller, 2004, Howren et al., 2009, Maes et al., 2009, Miller et al., 2009, Mossner et al., 2007, Raison et al., 2006, Schiepers et al., 2005). Notably, higher levels of IL-1β are known to affect synaptic transmission, causing impairments of learning and memory (Yirmiya and Goshen, 2011).

**Fig. 1:**
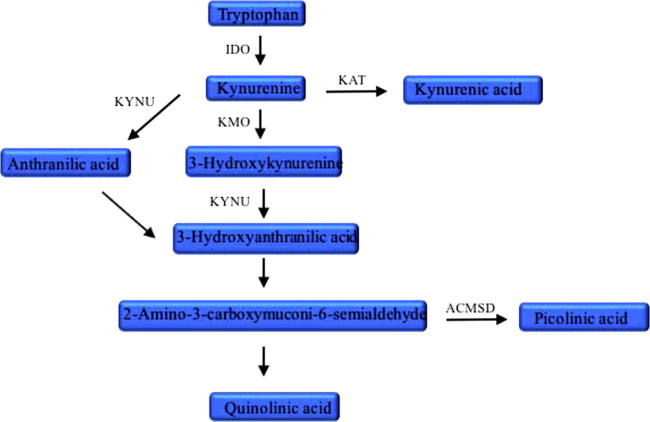
Simplified kynurenine pathway of tryptophan metabolism. ACMSD: aminocarboxymuconate semialdehyde decarboxylase, IDO: indolamine-2,3-dioxygenase, KAT: kynurenine aminotransferase, KMO: kynurenine 3-monooxygenase or kynurenine 3-hydroxylase, KYNU: kynureninase.

One of the underlying mechanisms by which IL-1β and other pro-inflammatory cytokines can affect neurogenesis is through alterations of the kynurenine pathway (see Fig. 1 for a simplified description). This pathway starts from the essential amino acid tryptophan and can generate the neurotoxic quinolinic acid (QUIN), a putative NMDA receptor antagonist (Schwarcz et al., 2012), whose accumulation can be prevented by production of picolinic acid (PIC), a potentially neuroprotective metabolite. Dysregulation in levels of several kynurenine pathway metabolites has been described in depression and other psychiatric disorders. For example, increased levels of QUIN have been detected in animal models of depressive-like behavior (Fischer et al., 2015), in hepatitis C patients with depression induced by IFN-α (Raison et al., 2010) and in the cerebrospinal fluid **(**CSF) of suicide attempters (Erhardt et al., 2013), who have also been shown to have reduced PIC and a decreased PIC/QUIN ratio in both CSF and blood (Brundin et al., 2016). Similarly, increased levels of kynurenine have been detected in depression induced by IFN-α (Capuron et al., 2003) and in the hippocampus of patients with Alzheimer’s disease (Guillemin et al., 2005), while the peripheral ratio of kynurenic acid/QUIN ratio has been shown to be reduced in depressed individuals (Meier et al., 2016, Savitz et al., 2015).

Considering the regulatory effects of these metabolites, the role of enzymes of the pathway that control their production is of significant interest. For example, inhibition of IDO, the first enzyme of the pathway, reduced the depression-like behavior induced by inflammation via peripheral (O'Connor et al., 2009) or intracerebral (Dobos et al., 2012) administration of lipopolysaccharide (LPS) in mice. Furthermore, inhibition of KMO reversed cognitive and motor deficits measured in neurodegeneration, using mouse models of Alzheimer’s disease and Huntington’s disease (Zwilling et al., 2011). Interestingly, KMO-deficient mice have been shown to be protected from inflammation-induced depressive-like behavior (Heisler and O'Connor, 2015, Parrott et al., 2016).

Our previous research has shown that IL-1β decreases neurogenesis while increasing levels of expression of all neurotoxicity-associated enzymes of the kynurenine pathway. This effect was partially reversed by KMO inhibition, suggesting that activation of the kynurenine pathway is indeed contributing to this cytokine-induced reduction of neurogenesis (Zunszain et al., 2012). Using the same *in vitro* model, we have also described how reduced neurogenesis induced by glucocorticoids is reversed by the SSRI sertraline (Anacker et al., 2011). Considering that IL-1β levels decrease in patients upon antidepressant treatment (Hannestad et al., 2011), we here explore the possibility that a regulation of neurogenesis in inflammatory conditions may be a common underlying pathway of antidepressant action. We investigate two antidepressants, the SSRI sertraline and the SNRI venlafaxine, as well as the two principal ω-3 polyunsaturated fatty acids components of fish oils, eicosapentanoic acid (EPA) and docosahexanoic acid (DHA), which have been associated with the pathogenesis and treatment of depression (Su et al., 2014, Su, 2012, Lin et al., 2012, Lu et al., 2010, Lin et al., 2010, Su, 2009). We have recently shown that these four compounds have different immune-modulating properties upon co-treatment with IL-1β: while all were associated with a corresponding decrease in the NF-kB activation caused by IL-1β alone, venlafaxine and EPA showed mostly anti-inflammatory properties but sertraline and DHA displayed mostly pro-inflammatory properties (Horowitz et al., 2015). In addition to looking at their effects on neurogenesis in the presence of an inflammatory insult, we here extend the study of their properties, evaluating additionally whether they regulate the metabolites and enzymes of the kynurenine pathway.

## 2 Materials and methods

### 2.1 Cell culture

All experiments were performed with the multipotent human hippocampal progenitor cell line HPC0A07/03C provided by ReNeuron (Surrey, UK). Cells were grown in reduced modified media consisting of Dulbecco’s Modified Eagle’s Media/F12 (DMEM:F12, Invitrogen, Paisley, UK) supplemented with 0.03% human albumin (Baxter Healthcare, Compton, UK), 100 µg/mL human apo-transferrin, 16.2 µg/mL human putrescine diHCl, 5 µg/mL human recombinant insulin, 60 ng/mL progesterone, 2 mM L-glutamine and 40 ng/mL sodium selenite. To maintain proliferation, 10 ng/mL human basic fibroblast growth factor (bFGF), 20 ng/mL human epidermal growth factor (EGF) and 100 nM 4-hydroxytamoxifen (4-OHT) were added. Cells were grown in 75 cm^2^ filtered cap culture flasks (Nunclon, Roskilde, Denmark) at 37 °C in 5% CO_2_ and regularly passaged at 80% confluence, until being transferred to plates for the differentiation experiments.

### 2.2 Assays with antidepressants and ω-3 fatty acids

To assess whether antidepressants and ω-3 fatty acids could abrogate changes in neuronal differentiation caused by IL-1β exposure, HPC0A07/03C cells were plated on 96 well plates (Nunclon) at a density of 1000 cells per well. Cells were cultured in the presence of EGF, bFGF and 4-OHT and treated with IL-1β (10 ng/mL) either alone or in combination with the antidepressant venlafaxine (1 μM) or sertraline (1 μM), or one of the ω-3 fatty acids, EPA (10 μM) or DHA (10 μM). Antidepressants and ω-3 fatty acids were dissolved in EtOH. All treatments had the same vehicle, including 0.1% ethanol, to exclude the possibility of any differences observed being the consequence of differing concentrations of solvents. Treatment conditions and doses were chosen as described in our previous work (Horowitz et al., 2015). After 3 days, cell-conditioned media was collected and stored at −80 °C for subsequent measurement of kynurenine metabolites, cells were washed and treatment with the cytokine and each of the four compounds was repeated in media without growth factors and 4-OHT for 7 subsequent days. At the end of the total incubation time (10 days), cells were fixed with 4% PFA to be further processed by immunocytochemistry. Treatment with the cytokine and all drugs was also conducted in 6 well plates to extract RNA after 24 h in proliferation media, in order to investigate changes in gene expression.

### 2.3 Immunocytochemistry

Neuronal differentiation and maturation were assessed by doublecortin (DCX) and microtubulin-associated protein-2 (MAP2) immunocytochemistry. Briefly, PFA-fixed cells were incubated in blocking solution (5% normal donkey serum, Scientific Laboratory Supplies Ltd, Nottingham, UK) in PBS containing 0.3% Triton-X for 2 h at room temperature, and with primary antibodies [rabbit anti-DCX, 1:500; mouse anti-MAP2 (HM), 1:500, Abcam, Cambridge, UK] at 4 °C overnight. Specificity of the MAP2 (HM) antibody for mature neurons in our cell culture was previously confirmed (Anacker et al., 2011). Cells were incubated sequentially in blocking solution for 30 min, secondary antibodies (Alexa 488 donkey anti-rabbit; 1:1000; Alexa donkey 555 anti-mouse, 1:1000, Invitrogen) for 1 h, and Hoechst 33342 dye (0.01 mg/ml, Invitrogen) for 5 min at room temperature. The number of DCX- and MAP2-positive cells over total Hoechst 33342 positive cells was counted with an automated approach using CellInsight NXT High Content Screening (HCS) Platform (ThermoScientific). Negative controls were incubated with unspecific mouse IgGs (1:500, control for MAP2), rabbit IgGs (1:500, control for DCX) in place of the specific primary antibody.

### 2.4 RNA isolation and cDNA synthesis

RNA was isolated using the RNeasy Micro Kit (Qiagen, Crawley, UK) following the manufacturer’s instructions and samples were kept frozen at −80 °C until further use. RNA quantity and quality were assessed by evaluation of the A260/280 and A260/230 ratios using a Nanodrop spectrometer (NanoDrop Technologies, Wilmington, DE, USA). Superscript III enzyme (Invitrogen, Carlsbad, CA, USA) was used to reverse transcribe 1 µg total RNA as previously described (Anacker et al., 2011).

### 2.5 Quantitative Real Time PCR (qPCR) analyses

qPCR was performed using Predesigned TaqMan Gene Expression Assay probes (Life Technologies) with TaqMan Universal PCR Master Mix with UNG (Life Technologies), using Chromo 4 DNA instrument from BioRad. The expression of target genes indoleamine 2,3-dioxygenase (IDO), kynurenine 3-monooxygenase (KMO), kynureninase (KYNU) and aminocarboxymuconate semialdehyde decarboxylase (ACMSD) was normalized to the expression levels of beta actin (ACTB) and glyceraldehyde-3-phosphate dehydrogenase (GAPDH) as references. The relative expression levels of target genes detected were calculated using the Pfaffl method (Bustin et al., 2005), with data normalized to the geometric mean of the housekeeping genes and expressed as fold change compared with the control sample. Three independent experiments were conducted on independent cultures, and each sample was tested in duplicate.

### 2.6 Sample preparation for metabolite analysis

After 3 days of proliferation, before media was changed to allow differentiation for 7 subsequent days, the cell media from each experimental condition was removed and centrifuged (2000 × rpm, 5 min) to pellet and remove dead cells. This supernatant was then assayed for metabolites of the kynurenine pathway, by adding an equal volume of ice cold 1 M perchloric acid (HClO_4_) fortified with deuterated tryptophan as internal standard (final concentration 1 µM); samples were centrifuged (15,000×*g*, 10 min at 4 °C) and the supernatants were collected for direct injection into the LC-MS/MS.

### 2.7 Liquid chromatography

The analyses of tryptophan (Trp), kynurenine (KYN), anthranilic acid (ANA), picolinic acid (PIC) and quinolinic acid (QUIN) were performed using an Agilent HP 1200 liquid chromatograph (Agilent, Milan, Italy) consisting of a binary pump, an autosampler and a thermostated column compartment. Chromatographic separations were carried out using a Discovery HS-F5 column (150 × 4.6 mm, 5 µm, Supelco, Milan, Italy) using 0.1% formic acid in water and acetonitrile (ACN) as mobile phase. The HPLC analyses were carried out using a linear elution profile of 15 min from 5% to 90% of ACN. The column was washed with 90% ACN for 3.5 min, then equilibrated for 5 min with 5% ACN. The flow rate was 0.5 mL/min. The injection volume was 40 µL. An Agilent 6410 triple quadrupole-mass spectrometer with an electrospray ion source operating in positive mode was used for detection. The optimized source parameters for MS analysis were: drying gas temperature 350 °C and gas flow 12 L/min, nebulizer gas flow pressure 35 psi and capillary voltage 4500 V. The optimized fragmentor voltage was 66 V and the collision energies were 4, 4, 8, 8, 8, 24 and 8 eV for Trp, KYN, ANA, PIC, QUIN and internal standard, respectively. The SRM pairs were 205–>188 (118), 209–>192 (94), 138–>120 (65), 124–>106 (51) and 168–>78 (114) and 210–>192 for Trp, KYN, ANA, PIC, QUIN and internal standard, respectively. The calibration curves were constructed using seven calibration standards and were linear over the concentration range of 0.001–2.000 µM for ANA, 0.01–5.00 µM for KYN, PIC and QUIN and 0.1–20 µM for TRP, with a correlation coefficient (r^2^) ≥ 0.9983 for all analytes. The limit of detection at a signal-to-noise ratio of 3:1 and lower limit of quantitation were 0.0005 and 0.0010 µM for ANA; 0.005 and 0.010 µM for KYN, PIC and QUIN; 0.05 and 0.1 µM for TRP.

### 2.8 Drugs and reagents

All drugs and reagents were purchased from Sigma–Aldrich (St Louis, MO, USA), unless otherwise stated. IL-1β, EGF and bFGF were purchased from Peprotech (London, UK).

### 2.9 Statistical analysis

Data are presented as mean ± SEM. All statistical analyses were performed with GraphPad Prism 7 on four independent biological replicates. One-Way ANOVA with Bonferroni’s post hoc test was used for multiple comparisons among treatment groups. Student’s *t*-test was used to compare means of two independent treatment groups; p-values of <0.05 were considered significant.

## 3 Results

### 3.1 Antidepressants and ω-3 fatty acids prevent the IL-1β-induced reduction of neurogenesis

**Fig. 2:**
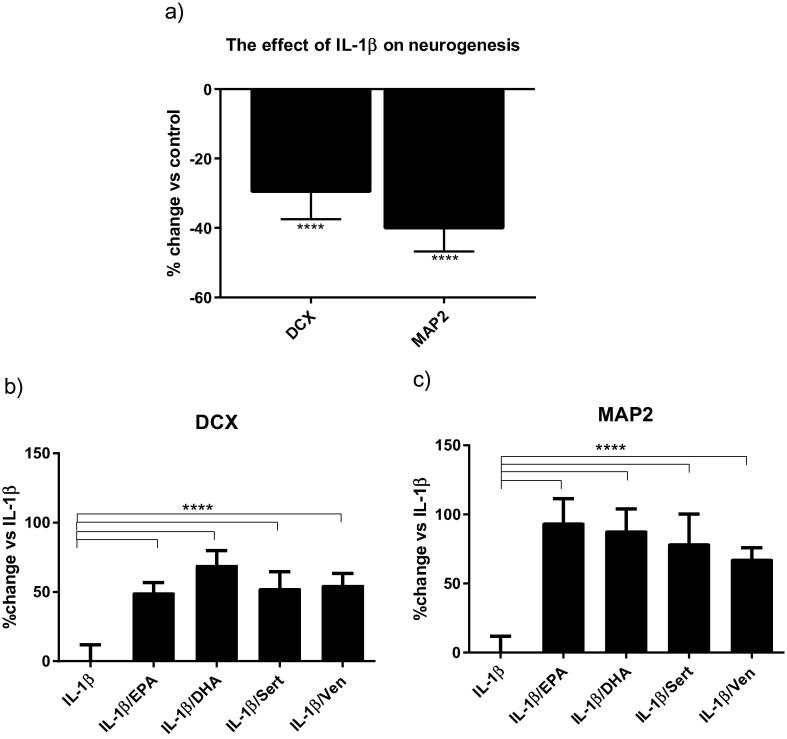
Monoaminergic antidepressants and ω-3 fatty acids reverse the IL-β-induced reduction of human hippocampal cells neurogenesis. Treatment with IL-1β decrease DCX*-*positive cells (immature neuronal phenotype), and MAP2 positive cells (mature neuronal phenotype) (a). Treatment with EPA, DHA, sertraline or venlafaxine reverse the IL-1β-induced decrease in DCX positive cells (b), and MAP2-positive cells (c); ^****^p < 0.0001.

Treatment of cells with IL-1β (10 ng/mL) for three days of proliferation followed by seven days of differentiation reduced the percentage of DCX-positive and MAP2-positive and cells by 28% and 40%, respectively (Fig.2a). These changes were similar to those we have previously reported (Zunszain et al., 2012). To establish whether the ω-3 fatty acids EPA and DHA, and the monoaminergic antidepressants venlafaxine and sertraline, were able to reverse these detrimental effects of IL-1β cells were co-incubated with the cytokine and each of the compounds at doses previously established (Horowitz et al., 2015). Co-incubation of cells with IL-1β and either EPA (10 μM), DHA (10 μM), sertraline (1 μM) or venlafaxine (1 μM) reversed the decrease in the percentage of DCX-positive neurons caused by IL-1β to +50%, +70%, +53% and +53%, respectively (Fig.2b). Of note, individual treatment with either ω-3 fatty acids or antidepressants did not affect the number of DCX-positive cells (data not shown). Similarly, the reduction of MAP2-positive cells caused by IL-1β was reversed upon co-incubation with all drugs to +94% with EPA, +88% with DHA, +79% with sertraline and +67% with venlafaxine (Fig.2c). Treatment with ω-3 fatty acids alone did not affect the number of MAP2-positive cells. In accordance with our previous data (Anacker et al., 2011), sertraline significantly increased the number of MAP2-positive cells by +7%, when compared with control (data not shown). Venlafaxine also enhanced MAP2-positive staining by +8% (data not shown). Although antidepressants alone were able to affect the number of MAP2-positive cells, this effect was much lower than what we report upon co-incubation with IL-1β and both antidepressants (+79% for sertraline and +67% for venlafaxine). Therefore, these findings are consistent with the notion that antidepressant compounds may play an important role in reversing the negative effects of inflammation on neurogenesis.

### 3.2 IL-1β treatment modulates levels of metabolites and enzymes of the kynurenine pathway

**Fig. 3:**
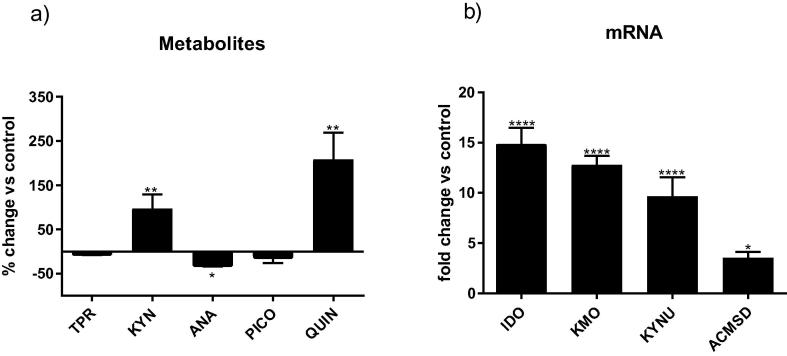
IL-1β modulates the production of metabolites and enzymes of the kynurenine pathway. Treatment with IL-1β affected the kynurenine pathway by (a) increasing levels of kynurenine and quinolinic acid and decreasing levels of anthranilic acid released into the supernatant, and (b) up-regulating the expression of IDO, KMO, KYNU and ACMSD; ^*^p < 0.05, ^**^p < 0.01, ^****^p < 0.0001.

Treatment of cells with IL-1β affected the production of metabolites and enzymes of the kynurenine pathway. Specifically, IL-1β increased kynurenine levels detected in the supernatant of proliferating cells exposed to the cytokine for 3 days by 96%, and increased levels of quinolinic acid by 208%, compared with vehicle-treated cells, and decreased levels of anthranilic acid by 28%. No significant changes were observed for either tryptophan or picolinic acid in the conditioned media (Fig.3a). Treatment with IL-1β increased levels of expression of enzymes involved in the production of quinolinic acid, namely IDO, KMO and KYNU, with fold changes of 15, 13 and 9, respectively, in line with what we have shown before (Zunszain et al., 2012). We here extended our evaluation of enzymes measuring ACMSD, which is responsible for limiting the formation of quinolinic acid by production of the neuroprotective metabolite picolinic acid. Treatment with the cytokine caused a 3-fold increase in levels of expression of this enzyme (Fig.3b).

### 3.3 Antidepressants and ω-3 fatty acids differentially modulate levels of metabolites of the kynurenine pathway

**Fig. 4:**
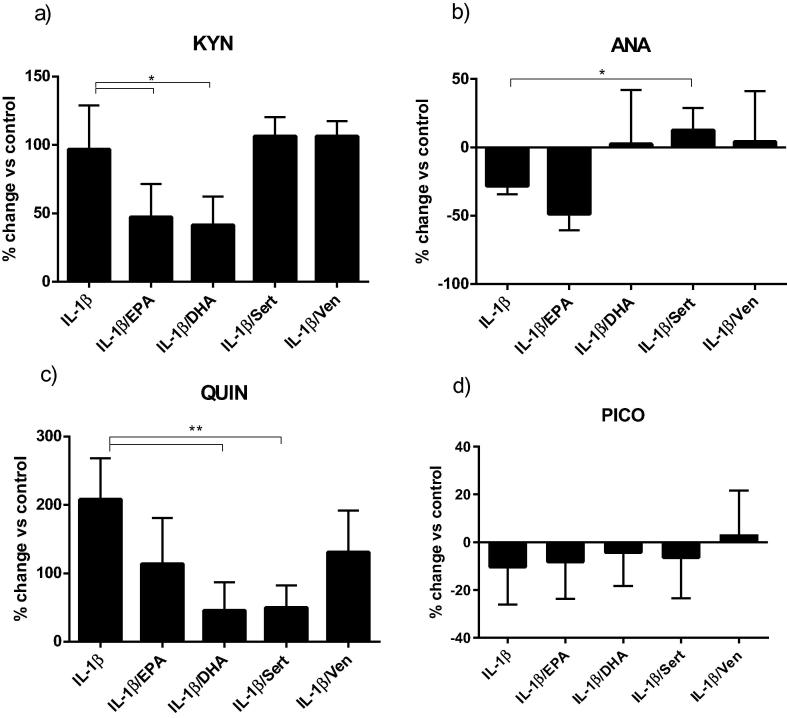
Antidepressants and ω-3 fatty acids modulate changes of kynurenine pathway metabolites caused by IL-1β treatment. Co-treatment of cells with IL-1β and ω-3 fatty acids partially abrogated the increase in kynurenine caused by the cytokine (a). Co-treatment with sertraline fully reverted the decrease in levels of ANA caused by IL-1β (b). Co-incubation with DHA and sertraline partially reverted the increase in QUIN levels caused by IL-1β (c). No significant changes in PIC were observed by any co-incubation (d); ^*^p < 0.05, ^**^p < 0.001.

To investigate whether the ability of antidepressants and ω-3 fatty acids to counteract the negative effects of IL-1β on neurogenesis involved effects on the kynurenine pathway, we first measured their action on metabolites. No compound significantly modulated the amount of tryptophan, highly present in the culture media, with respect to IL-1β exposed cells (data not shown), however the increase in levels of kynurenine caused by the cytokine was partially reverted by co-incubation with both ω-3 fatty acids (from 96% with IL-1β alone to 47% when co-incubated with EPA and 41% with DHA) (Fig.4a). On the contrary, the decrease in anthranilic acid was abrogated by co-treatment with sertraline (from −28% to +12%) (Fig.4b). Similarly, DHA and sertraline partially abrogated the rise in quinolinic acid caused by the cytokine (from +208% with IL-1β alone to +45% when co-incubated with DHA and +49% with sertraline), whereas EPA and venlafaxine showed a non-significant reduction in quinolinic acid levels (Fig.4c). There was no significant effect of either ω-3 fatty acids or antidepressants on levels of picolinic acid (Fig.4d).

### 3.4 Antidepressants and ω-3 fatty acids differentially regulate enzymes of the kynurenine pathway

**Fig. 5:**
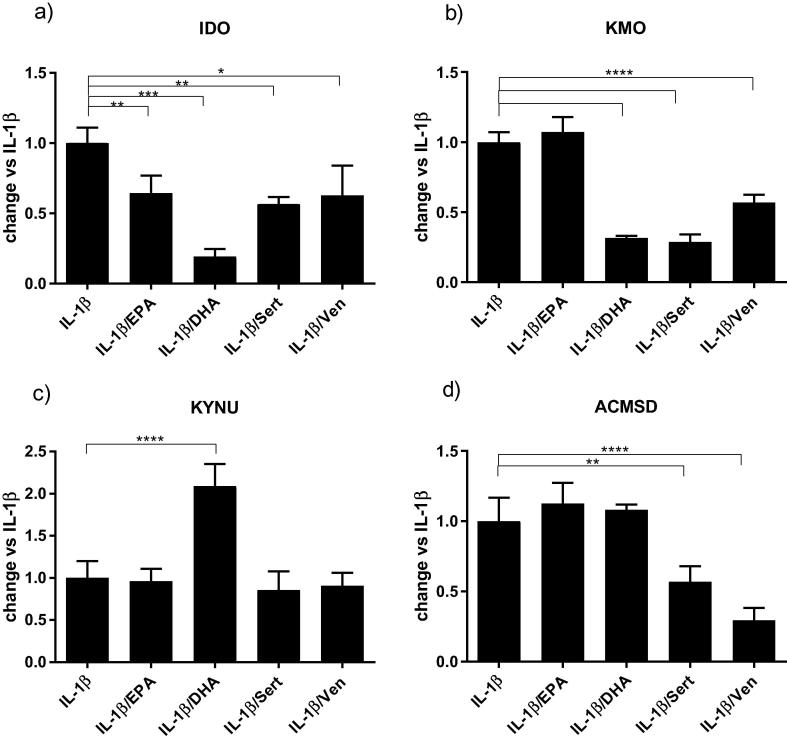
Antidepressants and ω-3 fatty acids modulate IL-1β-induced changes to the expression of enzymes of the kynurenine pathway. Co-treatment of cells with IL-1β and ω-3 fatty acids or antidepressants decreased the expression of IDO (a). Co-treatment with DHA, sertraline or venlafaxine decreased expression of KMO (b). Co-treatment with DHA upregulated KMO production (c). Co-incubation with sertraline or venlafaxine decreased the upregulation of ACMSD (d); ^*^p < 0.05, ^**^p < 0.01, ^***^p < 0.001, ^****^p < 0.0001.

Both antidepressants and ω-3 fatty acids altered the changes in mRNA levels of all the four enzymes of the kynurenine pathway that had been modulated by IL-1β exposure. EPA, DHA, sertraline and venlafaxine decreased the upregulation of IDO expression by 36%, 81%, 44% and 38% respectively, when compared with IL-1β alone (Fig.5a). Co-treatment with DHA, sertraline and venlafaxine decreased the upregulation of KMO caused by IL-1β by 69%, 72% and 44%, respectively (Fig.5b). Surprisingly, co-incubation with DHA caused a further 108% upregulation of KYNU when compared with IL-1β alone (Fig.5c). Finally, only sertraline and venlafaxine reduced the increased in ACMSD caused by the cytokine, with changes of 44% and 71%, respectively (Fig.5d).

## 4 Discussion

Our results show that EPA, DHA, sertraline and venlafaxine counteract the detrimental effects of IL-1β on our *in vitro* model of human hippocampal neurogenesis.

EPA, DHA, sertraline and venlafaxine lessened the reduction in DCX-positive neuroblasts and MAP2-positive neurons caused by IL-1β. This evidence that monoaminergic antidepressants and ω-3 fatty acids can reverse the anti-neurogenic effects of a pro-inflammatory cytokine on human hippocampal cells is of relevance given the importance attributed to immune dysfunctions in the pathogenesis of depression. Direct exposure to IL-1β or to its inducers, including LPS and IFN-α, has been shown to suppress hippocampal neurogenesis, along with the development of depressive-like symptoms in animal models (Goshen et al., 2008, Koo and Duman, 2008, Ekdahl et al., 2009, Monje et al., 2003, Kaneko et al., 2006). Nonetheless, despite these observations and work demonstrating that IL-1β is integral to the anhedonic and anti-neurogenic effects of stress (Koo and Duman, 2008), most evaluations of the protective effect of antidepressants in both animal and cellular experiments have modeled depression using stress paradigms or glucocorticoid exposure to simulate stressors. As such, chronic treatment with venlafaxine reversed the decrease in neurogenesis caused by chronic mild stress in mice (Zhang et al., 2015). Similarly, the suppressive effects of corticosterone on adult rat hippocampal cell proliferation were restored by paroxetine, another SSRI (Qiu et al., 2007). Additionally, we have shown that sertraline was able to restore the reductions of human hippocampal neurogenesis caused by *in vitro* treatment with dexamethasone in the same cellular model used for the present paper (Anacker et al., 2011). Only a few studies have used inflammatory models, aiming to efficiently mimic stress-related effects. Pre-treatment of human-like neuronal cells with trazodone, a tetracyclic antidepressant, completely reversed the decrease in cell viability caused by LPS and TNF-α (Daniele et al., 2015). Likewise, sertraline protected fetal rat hippocampal stem cells against LPS-induced cellular damage (Peng et al., 2012).

Inflammation has been thought to affect neurogenesis via several potential pathways: by activating the hypothalamic pituitary axis (HPA) axis to release cortisol and in turn reducing neurogenesis; by affecting glial cell function, regulating the production of neurotrophic factors; and by direct effects on neural progenitor cells (Song and Wang, 2011). The present *in vitro* study excludes systemic effects such as changes to the HPA axis, so it allows for the examination of direct effects upon progenitor cells. DHA and sertraline reversed the effect of IL-1β on reducing neurogenesis while also decreasing the production of quinolinic acid. At the same time, they caused a down-regulation of KMO mRNA expression. Hence, the ability of these compounds to block stimulation of quinolinic acid production or release induced by IL-1β may be the mechanism by which they block the cytokine-induced reduction in neurogenesis. Surprisingly, ACMSD, known to enhance the production of the neuroprotective picolinic acid, was significantly upregulated by IL-1β but also by co-treatment with IL-1β and EPA or DHA, perhaps suggesting the inductions of a counteracting mechanisms against the increase in quinolinic acid previously reported. However, the levels of the picolinic acid were not affected in our experiments. This might suggest that either the increase in ACMSD was not strong enough to induce an effect on the metabolite level, or that perhaps a change in ACMSD gene expression requires a longer time to modulate levels of picolinic acid. While activation of the kynurenine pathway can lead to a reduction in serotonin, the behavioral and neural effects observed upon inflammatory insults are believed to be primarily dependent on the formation of neurotoxic metabolites (O'Connor et al., 2009, Dantzer et al., 2011). In particular, quinolinic acid can increase neurotoxicity by various pathways, most notably by over-activation of NMDA receptors and increasing cytosolic calcium ions, leading to mitochondrial dysfunction, cytochrome *c* release, ATP exhaustion, free radical formation and oxidative damage (Perez-De La Cruz et al., 2012, Guillemin, 2012). Studies support the role of antidepressants acting on kynurenine metabolites, though they present a mixed picture. Evaluations in humans have shown that the reduced kynurenic acid/kynurenine neuroprotective ratio observed in depressed patients increased significantly following successful treatment with monoaminergic antidepressants (Myint et al., 2007). Similarly, treatment of patients with escitalopram showed a reduction of their initially elevated 3-hydroxykynurenine and quinolinic acid levels (Halaris et al., 2015). However, no effect of monoaminergic antidepressants was observed on levels of metabolites secreted by blood from depressed patients stimulated *in vitro* by LPS (Krause et al., 2012). Likewise, evaluations in animals have shown that imipramine reduced the initially elevated kynurenine/tryptophan ratio in the hippocampus of rats exposed to LPS or chronic mild stress (Elgarf et al., 2014), but caused no metabolite changes in a related study where the depression-like behavior was induced by IFN-α (Fischer et al., 2015). The regulation of kynurenine enzymes has also been measured: fluoxetine had no effect on KMO mRNA levels when used to prevent the development of depressive-like behavior in a mouse model of cancer related fatigue (Norden et al., 2015), while agomelatine, a melatonergic antidepressant, reversed the LPS-dependent increase in KMO expression in the hippocampus of rats (Molteni et al., 2013). Though conflicting, these studies are encouraging and support the notion that metabolites and enzymes of the kynurenine pathway are potential significant targets for therapeutic intervention. Interestingly, a recent study demonstrated that both EPA and DHA could increase neuronal differentiation in rat neural progenitor cells, but seemed to do so by alternative mechanisms, acting on different regulating genes to induce cell cycle arrest and neuronal differentiation (Katakura et al., 2013). Further investigation of the effect of these fatty acids, as well as a study of the action of sertraline and venlafaxine on cell cycle genes would be instructive.

Our findings are particularly relevant in understanding some of the clinical evidence related to the use of fish oils, and support previous evidence showing a positive effect of treatment with fatty acids on neuroinflammation, particularly in the context of depression (Mingam et al., 2008, Delpech et al., 2015, Labrousse et al., 2012, Larrieu et al., 2014, Bazinet and Laye, 2014, Orr et al., 2013, Lotrich et al., 2013, Kiecolt-Glaser et al., 2007, Freeman and Rapaport, 2011, Mocking et al., 2017). Recent meta-analyses suggested that EPA has better antidepressant effects than DHA in combination with antidepressant medications (Mocking et al., 2016, Grosso et al., 2014). Indeed, the clinical trials recruiting depressed patients taking antidepressant agents as a combination showed that either DHA is ineffective (Grenyer et al., 2007, Silvers et al., 2005) or EPA is more effective than DHA (Mozaffari-Khosravi et al., 2013). In addition, it has been reported that the combined effect of higher dose of EPA with lower dose of DHA was effective in reducing depressive symptoms in depression (Rondanelli et al., 2010, Rizzo et al., 2012) whereas the opposite combination (lower EPA and higher DHA) was ineffective (Silvers and et al., 2005, Su et al., 2003, Grenyer and et al., 2007), perhaps suggesting that the dosage, more than the type of treatment, might influence patients’ response. Our present work shows that treatment with either DHA or EPA has a positive effect on neurogenesis, with DHA also decreasing the production of the neurotoxic quinolinic acid. In contrast, in our previous study we demonstrated an overall anti-inflammatory pattern for EPA, but a mostly pro-inflammatory one for DHA upon co-treatment with IL-1β. Interestingly, both those effects were associated with a corresponding decrease in the NF-kB activation caused by IL-1β alone (Horowitz et al., 2015). In addition, we have also seen that EPA, but not DHA, can revert the damage caused by oxidative stress in our human hippocampal cell model. We have no indication about the overall status of the patients evaluated in clinical studies (including neurogenesis, oxidative stress, inflammation or regulation in the kynurenine pathway), so a possible overlap or the prominence of any of the possible dysregulations cannot be excluded. Considering that all clinical studies involved supplementation, it is also possible that EPA and DHA have differential interactions with various antidepressant drugs. Studies with individual combinations would therefore be of interest.

## 5 Conclusions

In summary, our observations indicate that venlafaxine, sertraline, EPA and DHA show protective effects against the detrimental influence of IL-1β on human hippocampal neurogenesis. We also find that one potential explanation for these effects is that these compounds may be involved in regulating IL-1β signaling by modulation of the kynurenine pathway, as DHA and sertraline reduce levels of the neurotoxic quinolinic acid increased by the cytokine, with a similar trend observed for EPA and venlafaxine. An increased understanding of the molecular mechanisms underlying these effects may allow for a more effective future personalization of antidepressant use.

## Conflict of interest

Professor Pariante and Dr. Zunszain have received research funding from Johnson & Johnson as part of a program of research on depression and inflammation, and research funding from the Medical Research Council (UK) and the Wellcome Trust for research on depression and inflammation as part of two large consortia that also include Johnson & Johnson, GSK and Lundbeck. The work presented in this paper is unrelated to this funding. All other authors declare no conflict of interest.
